# Improving knowledge about breast cancer and breast self examination in female Nigerian adolescents using peer education: a pre-post interventional study

**DOI:** 10.1186/s12905-021-01466-3

**Published:** 2021-09-10

**Authors:** Ayebo E. Sadoh, Clement Osime, Damian U. Nwaneri, Bamidele C. Ogboghodo, Charles O. Eregie, Osawaru Oviawe

**Affiliations:** 1 Institute Of Child Health, University Of Benin, PMB 1154, Benin City, Nigeria; 2 Department of Surgery, University Of Benin, PMB 1154, Benin City, Nigeria

**Keywords:** Breast cancer, Breast self examination, Knowledge, Female, Adolescents, Peer Education

## Abstract

**Background:**

Prevention of BC of which the cornerstone is creating awareness and early detection is important in adolescents and young women because of their worse outcomes. Early detection strategies such as mammography are currently beyond the reach of most women in sub-Saharan Africa.. Lack of awareness and late presentation contribute to the poor outcomes. Awareness creation among adolescents may result in modification of some risk factors for BC with adoption of healthy life styles including accessing early detection activities. This study determined the effect of peer education as a strategy to create awareness on BC and breast self examination (BSE) among in-school female adolescents in Benin City.

**Methods:**

This was a pre-post interventional study carried out in October –December 2016 on female students of four secondary schools in Benin City. Pre-peer training, using a pre-tested self-administered questionnaire, knowledge about BC and BSE was assessed in about 30% of each school population. This was followed by training of 124 students selected from the schools (one student per class) as peer trainers. The peer trainers provided training on BC and BSE (the intervention) for their classmates. Within two weeks of peer training knowledge about BC and BSE was reassessed in 30% of each school population. Selection of students for assessment pre and post intervention was by systematic sampling. Correct knowledge was scored and presented as percentages. Chi square test, student t test and ANOVA were used to assess associations and test differences with level of significance set at p < 0.05.

**Results:**

There were 1337 and 1201 students who responded to the pre and post-training questionnaires respectively. The mean BC knowledge score (20.61 ± 13.4) prior to training was low and it statistically significantly improved to 55.93 ± 10.86 following training p < 0.0001 Following peer training, statistically significant improvement (p 0.037- < 0.001) occurred in most knowledge domains apart from symptomatology. Pre-peer training 906(67.8%) students knew about BSE but only 67(4.8%). Significantly more students 1134(94.7%) knew about BSE following peer training.

**Conclusions:**

Peer education strategy can be used to improve BC and BSE knowledge in adolescents. This strategy is low cost and could be very useful in low resource settings.

**Supplementary Information:**

The online version contains supplementary material available at 10.1186/s12905-021-01466-3.

## Background

Breast cancer (BC) is the commonest cancer in women with an estimated 2.09 million new cases and 626,679 deaths reported in 2018 [1]. It is also the commonest cause of death from a malignancy among women in developing countries [1]. In Nigeria the age-standardized incidence rate of breast cancer ranges from 43.6 to 56.7/100,000 with age-standardized mortality rates of 16.7–20/100,000 [1].

Though commoner in older women, BC is reportedly responsible for 14% of all cancers in adolescents and young women (15-39 years) with poorer outcomes in this group. [2] In a Nigerian study, 28% of women accessing a radiotherapy service for breast cancer were aged below 40 years [3]. In developing countries the risk of death from BC is higher due to late presentation in hospital. In a report by Agbo et al.[4] from Sokoto in Nigeria, 99.4% of patients presented in late stage disease (stage 3c and stage4) whereas a systematic review reported a symptom duration of 8-12 months [5]. At such late stages only palliative care can be offered.

Late presentation has been linked to lack of awareness about BC [6]. In a systematic review on knowledge about BC in Nigeria, good knowledge was reported in majority of studies, however awareness about breast self examination (BSE) was low while its practice was even lower [7]. Few studies on BC and BSE are focused on adolescents probably because the prevalence of BC is low in this group [4]. Of the two studies focused on secondary school students in Nigeria, overall knowledge about BC was considered poor among the students even though awareness was high [8, 9]. Students in secondary schools cover most of the age range of adolescence.

The risk factors for BC include non-modifiable factors like age, genetics, race, parity, and breastfeeding while modifiable risk factors include alcohol intake and lack of physical activities [10]. Some of these risk factors for BC such as obesity, alcohol intake and lack of physical exercise involve life styles which could be modified or prevented early in life by pre-knowledge.

BC control through modification of potential risk factors could impact long term incidence of BC [11]. Early detection to improve outcomes and survival is a major cornerstone of BC control [11, 12]. Early detection and diagnostic mechanisms include BSE, clinical breast examination and mammography. Uptake of these interventions is very low especially in developing countries due to lack of awareness [7, 13]. Lack of access to health care (due to cost and geographic restriction of centres that offer the services to urban areas), prohibitive cost of mammography and inadequate numbers of specialist to carry out mammographic screening are barriers to optimal uptake of these interventions [14]. So BSE is considered a culturally acceptable and religion- friendly way of improving detection rates at no cost [15].

There has been recent calls for timely diagnosis of symptomatic BC which will result in clinical downstaging (that is detecting symptomatic BC at earlier stages) especially In populations where late presentation is common [16]. It has been shown that in these settings, improvement in detection even in the absence of mammography can provide substantial benefits [17].

Peer education has been used successfully in improving knowledge and changing behavior of adolescents especially in the area of reproductive health[18, 19] In India two strategies were compared; it was found that the child-to-child strategy( a form of peer education) was better at improving general knowledge on cancer than routine lectures [20]. Also some studies have shown that peer education is effective in improving knowledge and practice of BSE only but the target population were women/adolescents older than 18 years [21, 22]] In Nigeria, the studies on BC and BSE in adolescents recommend interventions to improve knowledge and practice. There is a paucity of studies on improving knowledge about BC and BSE in this target age group. One such study did not use the peer education strategy. [23] In this study, our aim was to determine if peer education can improve the knowledge of in-school female adolescents with respect to BC and BSE respectively.

## Methods

This pre-post intervention study was carried out between October and December 2016 as part of a larger study to improve the knowledge of female secondary school students on the two commonest cancers in women BC and cervical cancer. The study was carried out in Benin City which is the capital of Edo state in the South-South geo- political zone of Nigeria. It is largely urban. The methodology has been reported in an earlier publication and is summarized below [24].

### Ethical consent

The Research and Ethics Committee (REC) of the College of Medical Sciences, University of Benin provided ethical clearance for the study with REC approval number CMS/REC/2016/003. The Principals of the schools that participated in the study gave permission for the study to be carried out in their schools. Verbal consent/assent was obtained from the participating students. The purpose of the research was explained to all the students. The procedure for the research was described and they were told that participation was voluntary and withdrawal at any stage of the research was acceptable. No incentives were offered. The students were given opportunity to ask questions before the students were selected according to the protocol of the study and questionnaires distributed. The questionnaires had no identifiers.

### Study participants

#### Pre-peer training

The study recruited female students from four secondary schools in Benin City. About 30% of each school’s population of students was randomly selected by systematic sampling and baseline information obtained from them. The first student in each class was randomly selected and then every third student thereafter was picked. If a selected student refused to participate the very next student was used to replace her. The information was obtained using Self-administered questionnaires (see Additional file 1) that were distributed to the students during break period. Immediately after completion the questionnaires were retrieved.

#### Training of peer trainers

One week after the baseline information was collected, students from the different classes in both junior and secondary schools were selected to attend a training seminar on BC and BSE. These students who were to be the peer trainers were selected by the schools, one student per class. Most classes had several arms and each arm presented a student. CO, one of the authors gave a lecture on BC and BSE during which key information on BC and BSE (such as definition, risk factors, signs, symptoms, screening and diagnostic methods, treatment and prevention of breast cancer) was emphasized. Demonstration of how to conduct BSE was also carried out. At the seminar, pre and post test assessments of knowledge about BC and BSE were done using the same questionnaire that was used for collecting baseline data.

Each student (now peer trainer) was then given a flier containing the key information.

#### Peer training (the intervention)

Using the flier as a guide and within two weeks of the training, each peer trainer delivered a mini lecture on the subject to her classmates. The key points in the fliers were also emphasized.

#### Post-peer training assessment (Post intervention assessment)

Following delivery of the lectures by the peer trainers, an assessment on knowledge about BC and BSE was carried out in about 30% of the school student population using the same questionnaire. The assessment was done within a week of the lectures by the peer trainers. The first student in each class was randomly selected while every third student was subsequently selected (systematic sampling). The assessment was carried out during break period. The students in the school cohort who responded to the pre-peer training assessment were not exactly the same as those who responded to the post-peer training assessment. The questionnaires are thus not paired. Also the pre- and post- test questionnaires for the peer trainers were not paired.

### Study instrument

The same questionnaire was used for the collection of data at all points of evaluation – pre-peer training, pre- and post-test evaluations of peer trainers at the seminar and post-peer training in the schools. The questionnaire sought demographic information such as age, class, maternal and paternal levels of education and religion. Questions to ascertain knowledge about BC, its risk factors, symptoms, treatment and different aspects of BSE were also in the questionnaire. The questionnaire was developed after extensive literature review. Content validity was ascertained by expert review. The questionnaire was pretested at a secondary school that was not part of the study. Following pretesting some of the questions were re-phrased for better clarity while others were removed.

Correct answers to questions on knowledge were awarded one point each while zero was awarded to non-response, wrong response or don’t know response. Total scores were converted to percentages.

### Data analysis

The data obtained was entered into an IBM Statistical Package for Social Sciences ( SPSS) version 20 spread sheet. Analysis was carried out using the same soft ware and Graphpad Instat. The students who responded to the questionnaires in school are referred to as the school cohort while those who received training at the seminar are called the peer trainers.

The numbers of students with a given response to questions on the questionnaire were recorded as simple percentages. Fishers Exact test and Chi-squared test were used to test the association between variables. The knowledge of students was compared by calculating their mean knowledge scores.. Analysis of Variance (ANOVA) was used to test the significance of the difference between multiple means. The level of significance was set at 0.05 at 95% confidence level.

## Results

There were 124 students trained as peer trainers whereas the school cohort had 1337 who responded to the pre peer training assessment and 1201 who responded to the post peer training assessment. The mean age (Standard Deviation) of the peer trainers was 13.7(1.7) years while that of the pre- peer training and post-peer training school cohort was 13.01(1.8) and 13.36(1.64) years respectively.

Of the 124 peer trainers 65(52.4%) were from Junior secondary school while 873(65.3%) and 724(60.3%) of the pre-peer training and post-peer training school cohort respectively were also from junior secondary school. The remaining students were from senior secondary school. Some 44(3.3%) and 25(2.1%) of the pre and post peer training school cohort did not indicate their type of school. Majority of the students were from the Christian faith (122(98.4%), 1214(90.8%) and 1190(99.1%) for the peer trainers, pre- peer and post-peer training school cohort respectively). The others were muslims.

### Knowledge about breast cancer

None of the peer trainers (prior to training) knew that BC was not exclusive to females, which was not significantly different from the finding in the pre-peer training school cohort. There was only marginal but statistically significant improvement in knowledge following training both for the peer trainers (p = 0.029) and post peer training (p = 0.039) in the school cohort. (Table 1) Many students 39(31.5%) peer trainers and 527(42.2%) in the pre-peer training school cohort were also not aware that BC could affect those who are younger than 20 years. There was no change in the proportion who knew BC could occur in those younger than 20 years in the peer trainers while there was a statistically significant increase to 848(70.6%) among the post-peer training school cohort (p = 0.000).

**Table 1:** Distribution of in-school adolescents by knowledge on breast cancer risk factors and symptoms pre- and post- training for the peer trainers and pre- and post- peer training for the school cohort

Knowledge domain	Proportion of adolescents with correct responses									
	Peer trainers					School cohort				
	Pre		Post		*p* value	Pre-peer		Post-peer		*p* value
	n	%	n	%		n	%	n	%	
BC affects only females	0	0	6	4.8	0.029	22	1.6	36	3	0.039
BC cannot affect those < 20 years	85	68.5	85	68.5	1.109	773	57.8	848	70.6	0.000
Breastfeeding is protective	29	23.4	113	91.1	0.000	502	37.5	1063	88.5	0.000
*Risk factors for BC*										
Lack of physical exercise is a RF	21	16.9	113	91.1	0.000	227	17	1091	90.8	0.000
Obesity is a RF	4	3.2	118	95.2	0.000	62	4.6	1146	95.4	0.000
Excess alcohol intake is a RF	1	0.8	118	95.2	0.000	19	1.4	1135	94.5	0.000
First pregnancy under 25 years is RF	61	49.2	107	86.3	0.000	743	55.6	1061	84.6	0.000
*BC symptoms*										
Pain in breast is first symptom	0	0	0	0		27	1.7	0	0	0.000
BC may mimic eczema	116	93.5	87	70.2	0.000	846	63.3	801	66.7	0.239
Breast mass not associated with weight loss is not BC	12	9.7	3	2.4	0.030	113	8.5	19	1.6	0.000
Bloody discharge associated with breast mass is always cancer	1	0.8	52	41.9	0.000	286	21.4	595	49.7	0.000

BC = Breast cancer, RF = risk factor

With regard to symptoms of breast cancer, majority of the peer trainers prior to training erroneously thought pain was the first symptom of BC and that a breast mass not associated with weight loss could not be BC. However this erroneous belief was not corrected with training and significantly worsened following peer training in the school cohort. Concerning BC mimicking eczema, majority of the peer trainers 116(93.5%) knew this pre-training but significantly fewer students knew this following training (p = 0.000). There was no significant change in knowledge concerning this among the school cohort following peer training. Majority of the peer trainers and school cohorts did not know that bloody discharge associated with breast mass is not always cancer. The proportion with correct knowledge about this improved significantly following training for the peer trainers and peer training in the school cohort. (p = 0.000. Table 1).

### Knowledge of risk factors

Of the peer trainers 29(23.4%) knew that breastfeeding was protective (Table 1). This proportion increased to 113(91.1%) following training p = 0.000. A similar statistically significant increase from 502(37.5%) to 1063(88.5%) occurred in the school cohort following peer training p = 0.000. Knowledge about other risk factors was low prior to training for both the peer trainers and school cohorts (Table 1). The improvement in knowledge following training was statistically significant for all the risk factors both for the peer trainers and the school cohort p = 0.000.

### Breast self examination

Many 83(66.9%) peer trainers and 906(67.8%) of the students in the school cohort had heard about BSE prior to the training (Table 2). There was a statistically significant improvement in the proportions that heard about it to 117(94.4%) peer trainers and 1134(94.7%) of the school cohort following training. None of the peer trainers and only 65(4.9%) students in the school cohort practiced SBE. Majority of the students believed only nurses and doctors should conduct breast examination. This misconception was corrected following training with 116(93.5%) peer trainers and 1120(93.3%) students in the school cohort knowing that breast examination is not the exclusive preserve of health care workers.

**Table 2:** Knowledge of In-school adolescents about detection of BC, BSE and treatment of BC

Knowledge domain	Proportion of adolescents with correct responses									
	Peer trainers (n = 124)					School cohort				
	Pre		Post		p value	Pre-peer (n = 1310)		Post-peer (n = 1201)		p value
	n	%	n	%		n	%	n	%	
*Detection of BC*										
BC can be detected by BSE	69	55.6	122	98.4	0.000	806	60.3	1176	97.9	0.000
BC can be detected by Mammo	19	15.3	112	90.3	0.000	559	41.8	1129	94	0.000
BC can be detected by blood test	38	30.6	22	17.7	0.000	843	63.1	166	13.8	0.000
*Breast self examination*										
Have heard about BSE	83	66.9	117	94.4	0.000	906	67.8	1134	94.7	0.000
Have been taught how to do BSE	0	0	118	95.2		64	4.9	1161	96.7	0.000
Perform BSE regularly	0	0				65	4.9			
Should only be done by nurses and doctors	0	0	116	93.5		24	1.8	1120	93.3	0.000
Should be done at least monthly	110	88.7	110	88.7	1.159	1209	90.4	1099	91.5	0.519
Can be done while taking a bath	103	83.1	103	83.1	1.134	870	65.1	1048	87.3	0.000
Best position for BSE is lying down	0	0	93	75.0		382	28.6	994	82.8	0.000
During BSE armpits should be examined	84	67.7	97	78.2	0.086	996	74.5	908	75.9	0.839
Raising arms up is part of BSE	67	54.0	117	94.4	0.000	840	62.8	1146	95.7	0.000
Can tell the difference between benign and malignant breast mass	0	0	4	3.2		235	17.6	47	3.9	0.000
*Treatment*										
Removal of the breast	124	100.0	124	100.0		1255	93.9	1198	99.8	0.000
Radiation	46	37.1	99	79.8	0.000	454	34.0	990	82.4	0.000
Antibiotics	49	39.5	99	79.8	0.000	617	46.1	985	82	0.000
Anticancer drugs	92	74.2	110	88.7	0.005	733	54.8	1065	88.7	0.000

BC = Breast cancer, BSE = Breast Self Examination, Mammo = Mammography

With regard to the frequency of SBE majority of the students 110(88.7%) peer trainers and 1209(90.4%) students in the school cohort knew that BSE should be done at least monthly. Majority were also aware that BSE can be done while taking a bath; 103(83.1%) peer trainers and 870(65.1%) students in the school cohort. However none of the peer trainers and only 382(28.6%) of the school cohort knew that the best position for SBE is lying down. These proportions statistically significantly increased following training (p = 0.000) Table 2.

### Knowledge about detection of breast cancer and treatment

Knowledge of students about BC detection methods is shown in Table 2.Prior to training, only 69(55.6%) peer trainers and 806(60.3%) students in the school cohort knew that BC can be detected by BSE. These proportions statistically significantly increased to 122(98.4%) peer trainers and 1176(97.6%) students in the school cohort respectively following training (p = 0.000). However, the erroneous belief that BSE could differentiate between benign and malignant cancer was prevalent and this persisted even after training both for peer trainers and in the school cohort.

The most widely known modality for treatment of BC was removal of the breast with all peer trainers and 1255(93.9%) students in the school cohort knowing this (Table 2). Correct knowledge about the other treatment modalities prior to training was seen in 46(37.1%) and 92(74.2%) for radiation and anticancer drugs respectively for the peer trainers and 454(34%) and 733(54.8%) respectively for radiation and anticancer drugs in the school cohort. The proportion of students with correct knowledge about radiation as a treatment modality increased to 99(79.8%) and 990(82.4%) in peer trainers and school cohort respectively. Similar increase in the proportion of students with correct knowledge of anticancer drugs to 110(88.7%) among peer trainers and 1065(88.7%) in school cohort respectively was also noted. Only 49(39.5%) and 617(46.1%) of the peer trainers and students in the school cohort respectively correctly identified that antibiotics are not a treatment modality for BC. These proportions increased to 99(79.8%) and 985(82%) of peer trainers and school cohort respectively.

The overall mean knowledge score for peer trainers and students in the school cohort statistically significantly increased from 19.61 ± 12.94 and 20.61 ± 13.4% respectively to 60.32 ± 8.78% and 55.93 ± 10.86% respectively following training (p = 0.000). The difference in pre-training mean knowledge scores between the different classes amongst peer trainers was not significantly different. There was statistically significant increase in the mean knowledge score of each class at post-training evaluation (p = 0.000). The differences in the post-training mean knowledge scores between the different classes were also not statistically significant p = 0.601.

In the school cohort, the difference between classes in pre-peer training mean knowledge scores was not statistically significant (p = 0.685). However there was statistically significant increase in mean knowledge scores of each of the classes following peer training (p = 0.000). The differences between the mean knowledge scores of the different classes following peer training were not statistically significant (p = 0.177).

## Discussion

In this study majority of the respondents thought that BC was exclusive to females and majority also felt that breast cancer does not affect those younger than 20 years. This is in keeping with findings in previous studies which highlight the fact that many women do not consider themselves at risk hence they do not carry out BSE [25, 26]. While the incidence of BC in the studied age group is low and even lower still among males, it is important that they be aware of the general risk as well as individual susceptibility. This is especially important as it has been shown that adolescents and young adult women are more likely to be diagnosed with stage III/IV disease and high grade tumour than older women [2].

Knowledge about risk factors was generally low. This is similar to the findings among adolescents in Sri Lanka and in a previous Nigerian study [8, 27]. Many of the students did not know about the protective effect of breastfeeding. Such knowledge is important for decisions on the choice of infant feeding later in life. Majority of the students also did not know that some life style choices were associated with higher risk of BC such as lack of physical activity, obesity and excessive alcohol intake. The training improved the knowledge about risk factors in both the peer trainers and school cohort. Such improved knowledge may result in appropriate life style choices.

Many of the adolescents in this study were not conversant with symptoms of breast cancer. They did not know that even though a mass is unassociated with weight loss it could still be cancer and that the presence of bloody discharge is not always cancer. Unfortunately it would seem that the training did not improve knowledge in this domain. Poor knowledge about the symptoms of breast cancer has previously been reported.

While majority of the adolescents knew that removal of the breast was a treatment modality, radiotherapy was not well known. However the training improved knowledge about other treatment modalities besides mastectomy. Many students erroneously believed that the use of antibiotics was a treatment modality for BC. This possibly is a reflection of the general attitude in society in which antibiotics are used for practically all ailments especially as they are easily obtained over the counter. Use of unhelpful therapy contributes to delay in appropriate health-seeking behaviour. Correction of this wrong impression as was achieved by the training is thus important not only to improve health-seeking behaviour following onset of symptoms but also in the reduction of misuse of antibiotics and the attendant problems such as emergence of resistance. In a study on creating awareness in Ghana, 49% of the control group thought breast cancer was an infection but this wrong belief was even worse in the intervention group [28].

Although about 70% of the students had heard about BSE, only about 60% knew that it could detect BC. Fewer still knew about mammography. These findings are similar to those in the Sri Lankan study [27]. The training improved knowledge. It is important that all females know about the screening tools available for BC so that they can access them. Mammography is recommended for older age group whereas studies have shown that BC is increasingly being diagnosed in younger women especially in sub Saharan Africa [16, 29]. Thus use of BSE is important for the younger age group and in settings such as Nigeria where other screening methods may be inaccessible. Improved knowledge may also modify health seeking behavior such as increased utilization of screening tools in the future. Although BSE has not been shown to reduce mortality from BC, the World Health Organisation recommends it in the context of raising awareness among those at risk [11]. Even in developed countries with well -organized mammography- based programmes it is still affirmed that women be familiar with how their breasts normally look and feel like and report any changes immediately to a health care provider [29]. It has been documented that a significant proportion of breast cancers are self-detected [30, 31]. In one such study among black women it was found that past performance of BSE was associated with four to five fold higher odds of self- detected BC than mammogram- detected [30]. This emphasizes the need for women to perform BSE in all settings.

Many of the students had some idea about how BSE is conducted even though majority had not been formally taught on how to do it. Thus many did not know about some important aspects of the examination such as examining the armpits, raising the arms and that the best position to carry out BSE is while lying down. These aspects were improved following training. These findings are similar to those of a study on peer education in Turkey [22].

Few students carry out BSE regularly which is similar to findings from Riyadh city in which only 3.4% of the studied students performed BSE on a monthly basis [32]. The disparity between knowledge about BSE and its practice as reported in this study has also been previously reported [33]. This study did not examine the reasons for low level of practice of BSE among these adolescents but the belief of many that breast examination should be carried out by the health care professionals could have been contributory. While clinical breast examination (breast examination carried out by a health care worker) is prioritized over BSE, it is pertinent to know that adolescents and indeed many Nigerian women do not have access to health care workers. The barriers include inadequate numbers of health care workers and cost as many do not have health insurance and have to pay out of pocket for any health service [14].

Mean knowledge scores prior to training were not significantly different between the different classes. The mean knowledge scores about BC were increased significantly following peer training. This finding is similar to findings in studies on the effect of peer education on BSE in Turkey and Egypt although these studies did not examine knowledge about BC and they targeted age groups above 18 years. [21, 22]The post-seminar training and post-peer training scores for the different classes were not significantly different. This means that the same knowledge can be transmitted to adolescents irrespective of their classes. This finding is consistent with findings in a Nigerian study which showed that knowledge about BC was not significantly associated with class [8].

Peer training improved most knowledge domains. However, knowledge domains that were not improved by training were also not improved by peer training. This observation is due to the fact that the post seminar evaluation was not done immediately after the seminar. This would have enabled the identification of the areas of difficulty with re-emphasis and an opportunity to clarify issues. Some studies have also reported lack of improvement in some knowledge domains following educational activities to improve knowledge about BC [28, 34]. One such study identified the reason to be the use of medical terminologies [34].

The impact of this intervention on knowledge is immediate as documented in this study and in other studies on peer education [21, 22]. Future benefits such as reduction of BC due to improved life style, early detection of BC due to improved BSE/ improved utilization of other screening tools and reduced morbidity/mortality from BC due to early detection and treatment may accrue from this intervention but the assessment of such benefit is beyond the scope of this study.

The strengths of this study include the large number of participants which makes the results more generalizable and the target age group studied. Many studies focus on just knowledge, attitude and practice without an intervention to improve the status quo. Some other studies focused on improving BSE without including knowledge on BC. The current study was more encompassing. The limitation of the study is the fact that the pre and post evaluations for the peer trainers and the school cohorts were not paired. This however does not invalidate the findings as pooled knowledge was evaluated and the study showed the ability of peer education to improve knowledge in the study population.

## Conclusion

Knowledge about BC and performance of BSE were low in the studied adolescents. Such poor knowledge contributes to failure to make appropriate choices to improve modifiable risks. It also leads to delayed presentation and poor outcomes in the event of BC. Peer training is a strategy that can be used to improve knowledge about BC and BSE among adolescents. The advantages include that it is a low cost strategy that can reach many adolescents with minimal resources. The impact of such training is likely to extend beyond the target adolescents to friends in other schools and to their families. Precautions have to be taken to ensure that the peer trainers understand the subject so that wrong information is not passed along.

## Supplementary Information

**Additional file 1** Breast cancer questionnaire- This is a copy of the questionnaire that was used for collection of data at all points of evaluation - baseline, pre- and post-test evaluations at the seminar and post-peer training in the schools.

## Data Availability

The data sets used for this study are available from the corresponding author on reasonable request.

## Abbreviations

ANOVA: Analysis of variance
BC: Breast cancer
BSE: Breast self examination
LOE: Level of education
Mammo: Mammography
SPSS: Statistical package for social sciences
REC: Research and ethics committee
RF: Risk factor
