# Supplementary material for: Improving knowledge about breast cancer and breast self examination in female Nigerian adolescents using peer education: a pre-post interventional study
Source: BMC Womens Health. 2021 Sep 10;21:328. doi: 10.1186/s12905-021-01466-3 (PMC8431890; doi:10.1186/s12905-021-01466-3)
Supplement: Supplementary file 1 — Additional file 1 Breast cancer questionnaire- This is a copy of the questionnaire that was used for collection of data at all points of evaluation - baseline, pre- and post-test evaluations at the seminar and post-peer training in the schools. [file 12905_2021_1466_MOESM1_ESM.docx]

QUESTIONNAIRE ON KNOWLEDGE ABOUT BREAST CANCER AND BREAST SELF EXAMINATION

This questionnaire is asking you questions about your knowledge about breast cancer and breast self examination. Please answer every question.

1. Age as at last birthday…………………….
2. What class are you in?……………………..
3. What is the level of education of your father?
4. No formal Education B. Primary C. Secondary D. Tertiary
5. What is the level of education of your mother?
6. No formal Education B. Primary C. Secondary D. Tertiary
7. What is your father’s occupation?.................................
8. What is your mother’s occupation?..................................
9. What is your religion?....................................
10. Breast cancer only affects females True [ ] False [ ] Don’t know [ ]
11. Breast cancer can not affect persons younger than 20 years True [ ] False [ ] Don’t know [ ]
12. Breastfeeding protects against breast cancer True [ ] False [ ] Don’t know [ ]
13. The following are risk factors for breast cancer
14. Lack of physical exercise True [ ] False [ ] Don’t know [ ]
15. Obesity True [ ] False [ ] Don’t know [ ]
16. Excess alcohol intake True [ ] False [ ] Don’t know [ ]
17. Having first pregnancy under the age of 25years True [ ] False [ ] Don’t know [ ]
18. Concerning breast cancer
19. Pain in the breast is usually the first symptom True [ ] False [ ] Don’t know [ ]
20. It may mimic eczema True [ ] False [ ] Don’t know [ ]
21. A breast mass is not cancer if it is not associated with weight loss True [ ] False [ ] Don’t know [ ]
22. Bloody nipple discharge associated with a breast mass is always cancer True [ ] False [ ] Don’t know [ ]
23. Breast cancer can be detected by the following methods
24. Breast Self Examination True [ ] False [ ] Don’t know [ ]
25. Mammography True [ ] False [ ] Don’t know [ ]
26. Blood test True [ ] False [ ] Don’t know [ ]
27. Concerning breast examination
28. It should only be done by the doctor or nurse True [ ] False [ ] Don’t know [ ]
29. It should be done at least once a month True [ ] False [ ] Don’t know [ ]
30. It can be done while taking your bath True [ ] False [ ] Don’t know [ ]
31. The best position for breast self examination is lying down on the bed with a pillow under the shoulder on the side to be examined. True [ ] False [ ] Don’t know [ ]
32. The armpit should also be examined when examining your breast True [ ] False [ ] Don’t know [ ]
33. Part of examining the breast includes raising your arms above your head True [ ] False [ ] Don’t know [ ]
34. Breast examination can always tell the difference between a benign mass and breast cancer True [ ] False [ ] Don’t know [ ]
35. Treatment for breast cancer includes
36. Removal of the affected breast True [ ] False [ ] Don’t know [ ]
37. Radiation True [ ] False [ ] Don’t know [ ]
38. Antibiotics True [ ] False [ ] Don’t know [ ]
39. Anti cancer drugs True [ ] False [ ] Don’t know [ ]
40. Concerning breast self examination
41. I have heard of it True [ ] False [ ] Don’t know [ ]
42. I have been taught to examine my breasts True [ ] False [ ] Don’t know [ ]
43. I examine my breasts regularly True [ ] False [ ] Don’t know [ ]
